# The spread of zoonotic *Thelazia callipaeda* in the Balkan area

**DOI:** 10.1186/1756-3305-7-352

**Published:** 2014-07-30

**Authors:** Adnan Hodžić, Maria Stefania Latrofa, Giada Annoscia, Amer Alić, Relja Beck, Riccardo Paolo Lia, Filipe Dantas-Torres, Domenico Otranto

**Affiliations:** Department of Parasitology and Invasive Diseases, Veterinary Faculty, University of Sarajevo, Sarajevo, Bosnia and Herzegovina; Dipartimento di Medicina Veterinaria, Università degli Studi di Bari, Bari, Valenzano, Italy; Department of Pathology, Veterinary Faculty, University of Sarajevo, Sarajevo, Bosnia and Herzegovina; Department for Bacteriology and Parasitology, Laboratory for Parasitology, Croatian Veterinary Institute, Zagreb, Croatia; Department of Immunology, Aggeu Magalhães Research Institute, Recife, Brazil

**Keywords:** *Thelazia callipaeda*, Red fox, Dog, Cat, Balkan, Bosnia and Herzegovina, Croatia, Balkan regions

## Abstract

**Background:**

*Thelazia callipaeda* (Spirurida, Thelaziidae), also known as “oriental eyeworm”, is a small nematode parasite that lives in the conjunctival sac of domestic and wild carnivores, rabbits and even humans, causing mild (e.g., conjunctivitis, epiphora, and ocular discharge) to severe (e.g., keratitis, and corneal ulcers) ocular disease. This study reports, for the first time, the occurrence of *T. callipaeda* infection in the Balkan regions (i.e., Bosnia and Herzegovina and Croatia), it provides genetic evidence on the origin of the infection in that area and discusses potential expansion pathways in the near future.

**Methods:**

This survey was conducted in two Western Balkan countries, Bosnia and Herzegovina and Croatia. At necropsy, from January 2011 to April 2014, a total of 184 carcasses of red foxes were examined throughout the study area and worms were collected from the conjunctival sac. In the same period, worms were also collected during clinical examination from the conjunctival sac of four dogs and a cat from Bosnia and Herzegovina and two dogs from Croatia. All nematodes collected were morphologically identified and molecularly characterized by sequencing of partial *cox*1 gene.

**Results:**

*T. callipaeda* was observed in 51 (27.71%) foxes and the highest prevalence (50.0%) was in the region of East Bosnia. Beside the 4 cases of hyperemia (7.84%), most of the infected animals had no signs of ocular infection (n = 47, 92.15%). A total of 417 adult nematodes collected (364 from foxes, 51 from dogs, 2 from cat) were morphologically and molecularly identified as *T. callipaeda* haplotype 1.

**Conclusion:**

This is the first report of autochthonous cases of *T. callipaeda* infection in red foxes, dogs and cat in Bosnia and Herzegovina and Croatia and data presented here suggest that reports of thelaziosis in other Balkan areas are, as yet, not diagnosed most likely due to the lack of awareness of practitioners. In addition, data regarding the spread of the infection in Europe over the last ten years suggests that an increasing pattern in the distribution of this disease in domestic and wild animals should be expected in the future.

## Background

*Thelazia callipaeda* (Spirurida, Thelaziidae) is known as the “oriental eyeworm” because of its occurrence in the Far Eastern Countries (i.e., Indonesia, Thailand, China, Korea, Myanmar, India and Japan) and in the extreme eastern end of the former Soviet Union (Khabarovsk region) [1], but this spirurid has also become endemic in Europe where it primarily infests dogs and cats [2]. Indeed, following the first description in dogs, cats and foxes in Italy [3, 4], *T. callipaeda* has been increasingly reported in western France (Dordogne area) [5, 6], Switzerland [7, 8], Spain [9], and Portugal [10–12]. This nematode infection induces from mild (e.g., conjunctivitis, epiphora, and ocular discharge) to severe (e.g., keratitis and corneal ulcers) ocular manifestations in animals [13], as well as in humans [14]. Human cases are usually associated with poor, rural communities with low health and socio-economic standards, where heavily affected domestic (i.e., dogs and cats) and wild carnivores (e.g., foxes) live in close vicinity with humans [14–17].

The apparent geographical expansion of this nematode in previously non-endemic countries of Europe has been also attributed to the dispersal of the infection with wild carnivores (i.e., foxes, beech martens and wolves) and rabbits, which are suitable hosts for this parasite and may easily move in neighbouring regions [18, 19]. Despite the fact that wild fauna play a role in maintaining and spreading of *T. callipaeda* among pets [18], the presence of canine thelaziosis in Europe is more likely a consequence of the increased mobility of dogs (e.g., hunting, international tourism), as well as due to increased vectors and parasite circulation [4, 9]. In contrast, cats are less frequently in contact with the vector and it may be assumed that reports of feline thelaziosis from practitioners are rare due to difficulties in inspecting their eyes [4]. Studies on the molecular characterization of partial cytochrome *c* oxidase subunit 1 (*cox*1) gene sequences demonstrated the circulation of a single haplotype (named as haplotype 1 [20]) among *T. callipaeda* specimens from Europe, in contrast to the existence of seven distinct haplotypes within isolates from Asia [20]. In addition, the expansion of this nematode is related to the occurrence of its vector, *Phortica variegata* (Drosophilidae, Steganinae), which is a lachrymophagous fly with a zoophilic behaviour [21, 22]. Studying the ecology and the widespread seasonal occurrence of this drosophilid fly in a highly endemic area of southern Italy [23], a desktop implementation of the Genetic Algorithm for Rule-Set Prediction anticipated that large areas of Europe were likely to represent suitable habitats for *P. variegata* and, therefore, for the expansion of thelaziosis. After about 10 years since the above predictive niche model was published [23], *T. callipaeda* has been found in many areas of Europe spotted in such estimation [7, 9, 24].

Meanwhile, large areas of the Balkan regions were shown to be suitable for the development of the vector, therefore, facilitating the spread of canine thelaziosis. According to predictive data, this study reports, for the first time, the occurrence of *T. callipaeda* infection in the Balkan regions (i.e., Bosnia and Herzegovina and Croatia), it provides genetic evidence on the origin of the infection in that area and discusses potential expansion pathways in the near future.

## Methods

### Collection of *T. callipaeda*from foxes

The survey was carried out in the entire territory of Bosnia and Herzegovina (B&H), which covers 51,209.2 km^2^ and it is situated in the western part of the Balkan Peninsula (43° 52' N, 18° 25' E). The central and eastern part of the country is characterized by mountains (up to over 2.000 m above sea level) with continental mountain climate, while the northeast is predominantly flatland with moderate continental climate. The southern part (Herzegovina) has a Mediterranean climate and dominant karst and plain topography. Mean annual precipitation ranges between 800 mm and 2.000 mm depending on geographic region and climate type.

About 43% of territory is forested mainly with oak and beech, while the remaining areas are conifers such as fir and pine. Fruits harvested are grapes, apples, pears, raspberries and plums. The fauna are considered to be among the most diverse in Europe, as a result of its ecological heterogeneity, geomorphologic, hydrological and eco climate diversity, and wildlife includes bears, foxes, wolves, wild boars, wildcats, hares, deer and badgers, amongst the most represented [25].

**Figure 1:**
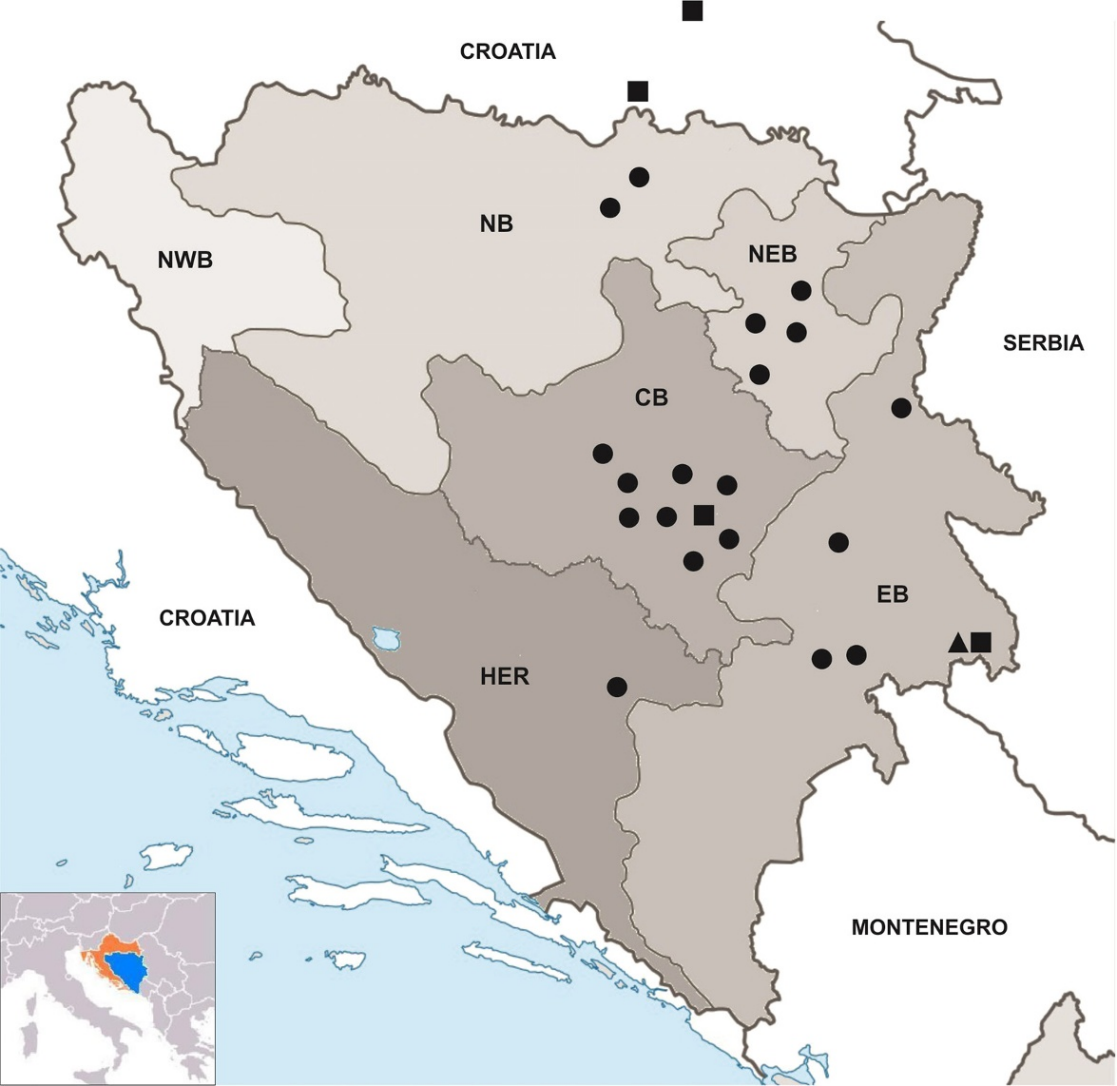
**Geographical distribution of** ***Thelazia callipaeda*** **-infected foxes (●), dogs (■) and cat (▲) in Bosnia and Herzegovina and Croatia.**

During the hunting season (October to March) between January 2011 and April 2014, carcasses of 184 red foxes (119 males and 65 females), mostly originating from coniferous forests in six different regions (Table 1, Figure 1), were collected as a part of an oral anti-rabies vaccination program throughout B&H. All animals were delivered to the Department of Pathology at the Veterinary Faculty in Sarajevo and stored in plastic bags at 4°C until necropsy. Data on sex, age, origin and ocular damage was recorded for each individual. At necropsy, adult nematodes were retrieved from the conjunctival sacs by flushing with saline solution.

**Table 1:** **Prevalence and geographical distribution of** *Thelazia callipaeda* **-infected foxes in Bosnia and Herzegovina**

Region	No. of examined foxes	*Thelazia callipaeda*-infected foxes	
		n	%
Central Bosnia (CB)	83	18	21.68
North Bosnia (NB)	8	2	25.00
North East Bosnia (NEB)	46	16	34.78
East Bosnia (EB)	26	13	50.00
North West Bosnia (NWB)	12	0	0.00
Herzegovina (HER)	9	2	22.22
Total	184	51	27.71

### Clinical cases in dogs and cat in the Balkan area

In order to investigate the spread of *T. callipaeda* in the Balkan area, a survey was conducted in B&H and Croatia (56,594 km^2^; 45° 80' N, 16° 13' E) as well, and dogs and a cat were also included. In October 2012 and April 2014, a 2-year-old male Labrador, an 11-year-old male Dalmatian from Sarajevo (43° 50' N, 18° 21' E) and two male hunting dogs and a female cat from Rudo (43° 37’ N, 19° 21’ E) were also referred to the Clinic of the Veterinary Faculty in Sarajevo, with clinical signs of unilateral or bilateral conjunctivitis. Animals were subjected to ocular examination after sedation and a few white worms were observed in the conjunctival sac. Eyeworms were then collected mechanically using sterile cotton swabs.

In December 2013 and January 2014, similar findings were made in two different municipalities in Croatia. Adult worms were found in conjunctival sacs of a 1-year and 4-months old mix breed female dog from Slavonski Brod (45° 10′ N, 18° 1′ E) and a 2-year-old male German Shepherd from Našice (45° 29′ N, 18°5′ E).

### Morphological and molecular identification

Nematodes collected from the eyes of the above animals were stored in 70% ethanol and sent to the Parasitological Unit of the Faculty of Veterinary Medicine (University of Bari, Italy) for morphological and molecular identification. A total number of 417 nematodes (364 from foxes, 51 from dogs, 2 from cat) were identified according to morphological keys [26] and kept in 70% ethanol until molecular processing. Briefly, males and females (7.5–13 mm and 12–18.5 mm in length and 340–430 μm and 370–510 μm in width, respectively) present striated cuticle on the entire body surface and a buccal capsule with a typically hexagonal oral opening. Males have a ventrally curved caudal end, 15 pairs of papillae on the ventral surface and two spicules, which greatly differ from each other in both shape and size [4]. In the female, the vagina opens anteriorly to the esophagus-intestinal junction and in the posterior half of the body. Immature eggs or germ cells are frequently observed (this is a differential characteristic with *Thelazia californiensis,* which presents the vagina opens posteriorly to that junction). The first stage larvae (L1, 382–400 μm long) present a shell membrane (i.e., embryonated eggs) and are arranged in a row in the distal uterus of the adult females [4].

Complete adult worms (n = 1–5, according to their sex, host geographical origin and total number collected from each animal) were morphologically identified and molecularly processed. Genomic DNA was extracted using a commercial kit (DNeasy Blood & Tissue Kit, Qiagen, GmbH, Hilden, Germany) and a partial sequence of the mitochondrial cytochrome *c* oxidase subunit 1 gene (*cox*1 - 689 bp) was amplified by PCR as previously described [20]. Sequences were determined in both directions (using the same primers individually as for the PCR) and the electropherograms verified visually. Sequences were aligned using the ClustalW program [27]. The alignments were verified by eye and compared with the sequences available in GenBanK (i.e., NCBI at http://www.ncbi.nlm.nih.gov/) for the *cox*1 of *T. callipaeda*.

### Statistical analyses

Animal data collected were analysed using SPSS 17.0 statistical software. A Chi-square test was used to test for associations between parameters. Differences were considered significant if *p* value was < 0.05.

### Ethical statement

The study was conducted under the frame of Project ID: BIH-PSD-G-EC 30, Sub project ID: CRIS Number: 2010/022-259, for the vaccination against rabies and in accordance with the veterinary law of B&H.

## Results

**Figure 2:**
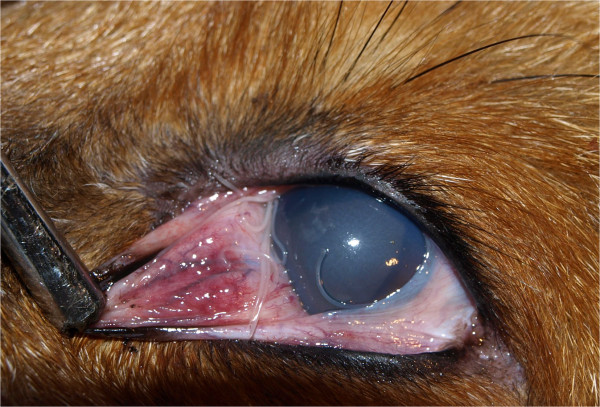
**Adult specimens of zoonotic** ***Thelazia callipaeda*** **in the left eye of a red fox.**

Out of the 184 red foxes examined, 51 (27.71%) were positive for *T. callipaeda*. Infection was recorded in 19 municipalities of 5 regions and the highest prevalence was detected in the region of East Bosnia (Table 1, Figure 1), differences observed in prevalence between regions were not significant (*p* = 0.154). Besides the 4 cases of hyperemia (7.84%), most of the infected animals had no signs of ocular infection (n = 47, 92.15%). A total of 364 adult worms were collected from the infected foxes, with a mean intensity (mean ± SD) of 8.08 ± 9.41 (Table 2). Adults were found in the left eye of 16 (31.37%), in the right eye of 14 (27.45%) and 21 (41.17%) foxes had worms in both eyes (Figure 2). Only 6 foxes (11.76%) harboured a single parasite. No significant differences were recorded in infected animals when compared with their sex (*p* = 0.608) or age (*p* = 0.708).

**Table 2:** **Number of** ***Thelazia callipaeda*** **-infected animals from Bosnia and Herzegovina and Croatia categorized by sex and age**

Host species	Host data					Nematode data					
	Male	Female	< 1 yr	> 1 yr	Total	Male	Female	Right eye	Left eye	Range (mean ± SD)	Total
Bosnia and Herzegovina											
Fox	31	20	5	46	51	ND	ND	186	178	1-50 (8.08 ± 9.41)	364
Dog	4	-	-	4	4	3	7	4	6	1-5 (2.5 ± 1.91)	10
Cat	-	1	-	1	1	-	2	-	2	-	2
Croatia											
Dog	1	1	-	2	2	ND	ND	ND	ND	13-28 (20.5 ± 10.6)	41
Total	37	22	5	53	58	3	9	190	186	1-50 (15.73 ± 9.38)	417

Number, sex and location of nematodes and mean intensity ± standard deviation (x ± SD) of infection are also included. (ND – no data).

Similarly, four dogs (10 worms) and one cat (2 worms) from B&H and two dogs (41 worms) from Croatia were positive for *T. callipaeda* (Table 2). All animals had moderate conjunctivitis, but severity of clinical signs was not related to worm burden. The locations of *T. callipaeda* infected foxes, dogs and cat from B&H and Croatia are shown in Table 1 and Figure 1. All specimens (n = 417) recovered were morphologically identified as *T. callipaeda* and the *cox*1 sequences obtained were shown to be identical to the sequence of *T. callipaeda* haplotype 1 (GenBank accession number AM042549).

## Discussion

Autochthonous cases of *T. callipaeda* infection have been reported for the first time in foxes, dogs and cat in B&H and Croatia. Foxes infected with *T. callipaeda* inhabit areas ranging from 126 to 1.201 m above sea level and came from almost all the geographical locations in B&H, indicating that the infection is endemic and widespread in the area. Among all the regions surveyed in this study, the highest prevalence was detected in the region of East Bosnia (50.0%). This area is characterized by mountains (500–2.000 m above sea level) with river streams and average annual precipitation ranging from 1.000 to 1.200 mm and the presence of orchards and deciduous woods, as well as by a rural environment with many hunting and stray dogs, thus making this region highly suitable for the vector. The latitudes of the two countries in our survey (between 43° and 45° North) fall within that of the Far Eastern and Asian countries (i.e., 10° and 45° North for India and Japan, respectively) where canine and human thelaziosis have for a long time been thought to be confined [4]. Moreover, these countries have the same latitude as those of some other endemic areas in Europe, namely, north-western Italy (Piedmont, latitude: 45° N; [4]) and France (latitude: 45° N; [5]). The climate and habitat conditions of the studied areas are similar to those above northern Italy and France, as the southern part of the country (Herzegovina) is more similar to those of southern Italy, where canine thelaziosis is highly endemic [4, 23]. Accordingly, all the areas under investigation fall within the provisional model for the distribution of *P. variegata* [23], indicating the power of such a geoclimatic model.

The high prevalence (27.71%) of thelaziosis reported in the examined foxes may indicate that this infection has spread only recently. This hypothesis is also supported by the fact that all the *cox*1 herein obtained were identified as the only haplotype (i.e., h1), which is the sole haplotype to be detected in domestic and wild animals in Europe [20], irrespective of the area of provenience and animal host from where they were collected.

Although the prevalence of the infection in foxes (27.71%) is lower than that previously found in a highly endemic area of southern Italy (49.3%; [19]), it is higher than that recorded in northern Italy (5.1%; [4]) and in Switzerland (5.7%; [8]), indicating the status of hyper-endemicity of the eyeworm infection in the study area. Indeed, data suggests a sylvatic cycle of thelaziosis occurs under natural conditions most likely due to the frequent contact of wildlife with the vector. Although other wildlife species were demonstrated to act as hosts for *T. callipaeda* (e.g. wolves*,* beech martens*,* brown hares, and wild cats) the role of foxes in spreading the infection has been discussed elsewhere [19]. Indeed, foxes are probably the most suitable hosts due to their habits, which may favor their contact with the vector. In spite of the scant scientific information on the biology, ecology and zoophilic habits of *P. variegata*, it is known that these flies usually fly early in the morning and late in the evening [22] when the foxes are active. Accordingly, the seasonality and the crepuscular activity of *P. variegata* coincide with the activity patterns of foxes that spend the majority of their active time during dawn and dusk [28]. The above considerations and the finding of a single haplotype of *T. callipaeda* throughout Europe also indicate a high degree of specificity of the nematode for its vector [20–22]. Red foxes are usually present with a low population density due to the fact that individual adults of the same sex distance themselves within own territories that can vary between 10 and 30 km [29, 30]. Therefore, foxes may have played a role in spreading thelaziosis migrating throughout the Alps in previously non-endemic areas where the vector was present. However, the role of other wild carnivores, such as wolves, as possible reservoirs and spreaders of thelaziosis should be investigated since, unlike foxes they occupy vast territories (even greater than 800 km) and they leave the pack to search for a new territory when they reach the reproductive age (22–24 months) [31].

Our results support the existence of a sylvatic life cycle of *T. callipaeda* and also further indicate that it is mainly maintained by foxes, but initial foci in non-endemic areas could also be due to “visiting dogs” and further settlement in foxes and other hosts being established as endemic in these areas. The present study also provides the first description of clinical cases of *T. callipaeda* infection in dogs and cat from the Balkan area and this finding should alert veterinary practitioners to include thelaziosis in differential diagnosis of ocular diseases, even in animals without travel history. This nematode is rather apparent in the animal eyes at the clinical examination of the conjunctiva, and its occurrence should not be misdiagnosed or overlooked.

## Conclusion

Data presented here suggests that reports of thelaziosis in other Balkan areas are, as yet, not diagnosed, most likely due to the limited awareness of practitioners. In addition, data regarding the spread of the infection in Europe over the last ten years suggests that an increasing pattern in the distribution of this disease in domestic and wild animals should be expected in the future.
